# Production of biologically active IL‐36 family cytokines through insertion of N‐terminal caspase cleavage motifs

**DOI:** 10.1002/2211-5463.12044

**Published:** 2016-03-12

**Authors:** Danielle M. Clancy, Conor M. Henry, Pavel B. Davidovich, Graeme P. Sullivan, Ekaterina Belotcerkovskaya, Seamus J. Martin

**Affiliations:** ^1^ Molecular Cell Biology LaboratoryDepartment of GeneticsThe Smurfit InstituteTrinity CollegeDublin 2Ireland; ^2^ Cellular Biotechnology LaboratorySaint‐Petersburg State Institute of TechnologyRussian Federation

**Keywords:** caspase, cell death, IL‐1 family, IL‐36, inflammation, protease

## Abstract

Recent evidence has strongly implicated IL‐36 cytokines as key initiators of inflammation in the skin barrier. IL‐36 cytokines belong to the extended IL‐1 family and, similar to most members of this family, are expressed as inactive precursors that require proteolytic processing for activation. Because the proteases responsible for activation of members of the IL‐36 subfamily have not been reported, we have developed a method for the production of biologically active IL‐36 through introduction of a caspase cleavage motif, DEVD, within the N‐termini of these cytokines. Here, we show that DEVD‐modified IL‐36α, IL‐36β and IL‐36γ cytokines were highly soluble and were readily processed and activated by caspase‐3. Caspase‐3‐processed IL‐36 family cytokines exhibited robust biological activity on a range of responsive cell types, including primary keratinocytes. We also generated specific polyclonal antibodies against all three IL‐36 family members through immunization with purified recombinant IL‐36 cytokines. The modified forms of IL‐36 described herein will be useful for production of large quantities of biologically active IL‐36 for structure and function studies on these important proinflammatory cytokines.

**Abbreviations**
Ac‐DEVD‐AFC: Ac‐Asp‐Glu‐Val‐Asp‐7‐amino‐4‐trifluoromethylcoumarin
DEVD: Asp‐Glu‐Val‐Asp
DMEM: dulbecco's modified eagle's medium
ER: endoplasmic reticulum
GST: glutathione S‐transferase
IL‐1AcP: IL‐1 receptor accessory protein
IL‐36RA: IL‐36 receptor antagonist
IL‐36R: IL‐36 receptor
IPTG: isopropyl b‐D‐1‐thiogalactopyranoside
NFDM: non‐fat dry milk
Ni‐NTA: nickel‐nitrilotriacetic acid
PARP: poly (ADP‐ribose) polymerase
PMA: phorbol 12‐myristate 13‐acetate
poly(I:C): polyinosinic:polycytidylic acid
PRB: protease reaction buffer
RFU: relative fluorescence units
SEAP: secreted embryonic alkaline phosphatase
SUMO: small ubiquitin‐like modifier
TEV: tobacco etch virus
Ulp1: ubiquitin‐like‐specific protease 1

IL‐1 family cytokines, which include the recently described IL‐36α, IL‐36β and IL‐36γ proteins, play major roles as initiators of inflammation and are frequently among the first cytokines produced in response to infection or injury 1, 2, 3, 4. IL‐1 family cytokines can initiate complex cascades of additional cytokine production from diverse cells types 5, 6, 7, 8, 9, 10. IL‐36α, IL‐36β and IL‐36γ are encoded by distinct genes and accumulating evidence suggests that these cytokines play a key role in skin inflammation, particularly in psoriasis 11, 12, 13, 14, 15, 16, 17, 18, 19, 20. Members of the IL‐1 family are typically among the most upstream cytokines to be released upon infection or cell damage and most likely represent the key damage‐associated molecular patterns (DAMPs) as a consequence of their release during necrosis 4, 21.

IL‐36α, IL‐36β and IL‐36γ are all generated as leaderless cytokines that lack biological activity and are not secreted via the canonical endoplasmic reticulum (ER)–golgi secretion route 22. Thus, proteolytic processing of IL‐36 cytokines is required to unleash their proinflammatory activity, similar to other members of the IL‐1 family, such as IL‐1β and IL‐18 23, 24. Sims and colleagues have shown that removal of a small number of residues from the N‐termini of IL‐36α, IL‐36β and IL‐36γ increases their biological activity by greater than 10 000‐fold 22. However, the proteases responsible for activation of IL‐36 cytokines are currently unknown.

To facilitate investigations into the biological activity of the IL‐36 cytokine subfamily, a simple and efficient approach for the production of biologically active IL‐36 cytokines is desirable. Here, we describe a novel strategy for the production of large amounts of soluble active recombinant IL‐36 cytokines through insertion of a caspase‐3 cleavage motif (DEVD) within the N‐termini of these cytokines, followed by processing by purified recombinant caspase‐3. This approach enabled us to process and activate human IL‐36 cytokines with exquisite specificity, leading to a dramatic increase in biological activity. Here, we show that caspase‐3‐processed recombinant IL‐36 is highly active on a range of cell types, including primary keratinocytes, and elicits robust production of multiple cytokines and chemokines from target cells. We also established a convenient reporter cell line HeLa^IL‐36R^ overexpressing the IL‐36 receptor, as well as an NFκB‐SEAP reporter plasmid, which will be useful as a screening system to identify molecules involved in IL‐36 activation and downstream signalling. Using purified forms of IL‐36, we also generated polyclonal antibodies against all three IL‐36 cytokines that displayed specificity for individual IL‐36 proteins.

## Results

### Generation of DEVD‐modified IL‐36 cytokines

Previous studies have shown that the full‐length forms of IL‐36α, IL‐36β and IL‐36γ exhibit little or no bioactivity 22, similar to other members of the extended IL‐1 family, but become active upon removal of small stretches of N‐terminal amino acids. For example, truncation of IL‐36α after Leu5 has been shown to enhance its activity 10 000‐fold, similarly, truncation of IL‐36β after Gln4 and IL‐36γ after Gln17 also unleashed the activity of the latter cytokines 22. However, the proteases that naturally process at these sites to activate the latter cytokines have not been reported to date.

**Figure 1:**
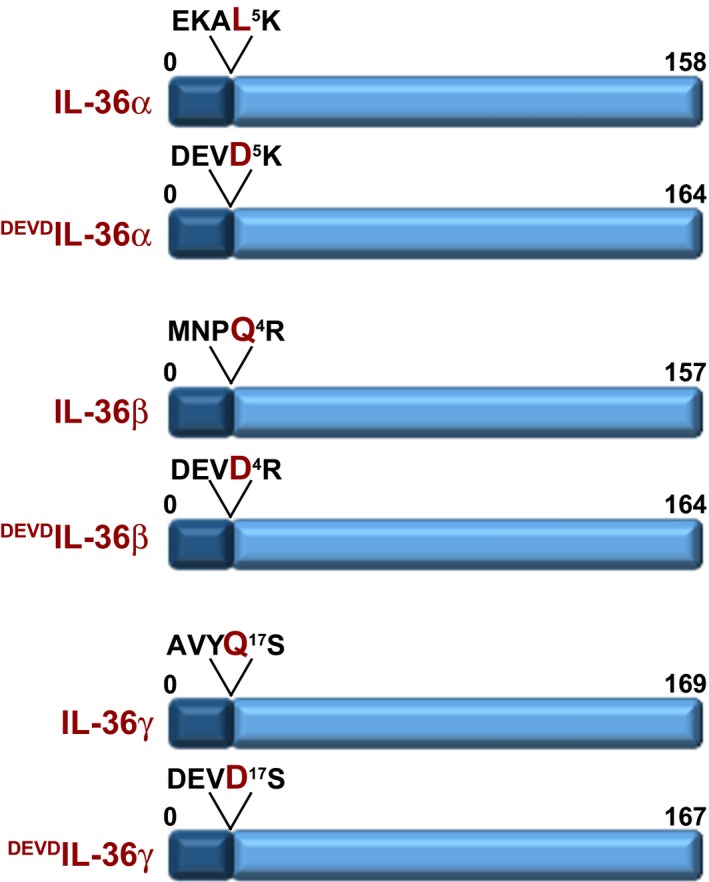
IL‐36 cytokines require processing to release biological activity. Schematic of modified forms of IL‐36α, IL‐36β and IL‐36γ where a caspase‐3‐processing motif (DEVD) was inserted into the IL‐36 sequence, N‐terminal to the known artificial processing sites 22.

To facilitate activation of recombinantly expressed IL‐36 cytokines for further study of their biological activity, we generated insertions within the N‐terminal region of all three IL‐36 coding sequences to insert the known caspase‐3 cleavage motif, DEVD (Fig. 1). Caspase‐3 is a relatively specific endoprotease that cleaves after aspartic acid (Asp) residues contained within specific tetrapeptide motifs that are not commonly found at spatially accessible sites within the majority of proteins 25, 26. Thus, insertion of caspase‐3 cleavage sites permits the generation of highly precise protein truncations to eliminate purification tags, autoinhibitory domains, or other unwanted protein domains prior to use in biological assays.

**Figure 2:**
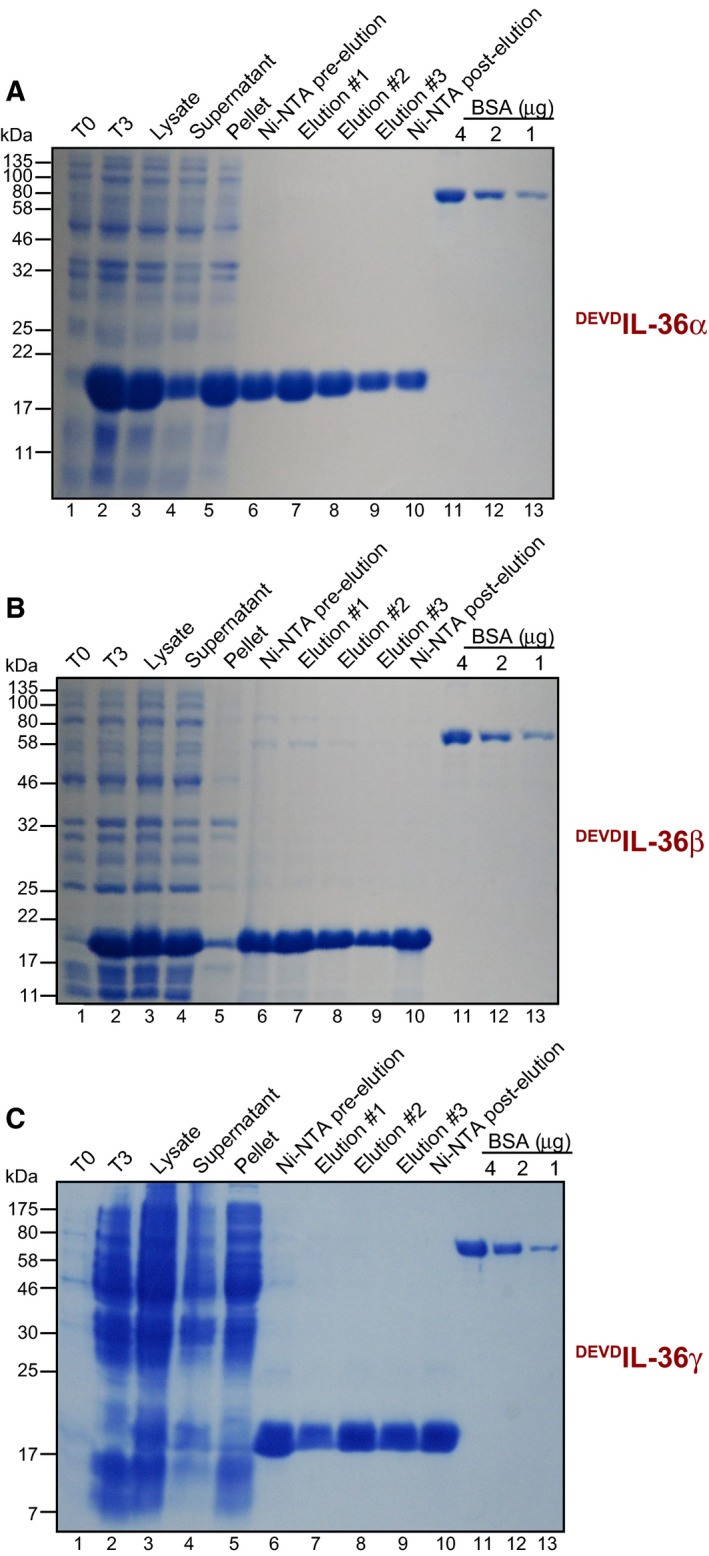
SDS/PAGE analysis of purified DEVD‐modified IL‐36. (A) SDS/PAGE gel of samples taken at the indicated stages of bacterial expression and purification of ^DEVD^IL‐36α from a 250 mL culture of BL21‐CodonPlus(DE3)‐RIL bacteria. Samples of bacterial lysate were taken before (T_0_) (lane 1), or after (T_3_) (lane 2), induction of protein expression. Note the appearance of the ~18 kDa ^DEVD^IL‐36α band after induction of expression. Equivalent volumes of bacterial lysate after sonication (Lysate) (lane 3), the clarified bacterial supernatant (Supernatant) (lane 4) and the insoluble material removed (Pellet) (lane 5) were also run. A sample of Ni‐NTA agarose before and after protein capture was run alongside 10 μL samples of each elution fraction. (B) SDS/PAGE gel of samples taken at various stages of bacterial expression and purification of ^DEVD^IL‐36β from a 250 mL culture of BL21‐CodonPlus(DE3)‐RIL bacteria. (C) SDS/PAGE gel of samples taken at various stages of bacterial expression and purification of ^DEVD^IL‐36γ from a 250 mL culture of BL21‐CodonPlus(DE3)‐RIL bacteria. 10 μL samples of each elution fraction were run. Samples were run alongside indicated amounts of BSA for estimation of protein concentration. Molecular weight markers are shown (kDa).

The DEVD motifs were inserted at regions within IL‐36 cytokines previously identified by Sims and colleagues to result in the liberation of robust biological activity upon proteolysis at these sites 22. We then transformed BL‐21 (RIL) strain *E. coli* with pET45b‐based IL‐36 expression plasmids, followed by induction of protein expression for 3 h at 37 °C. As shown in Fig. 2, all three IL‐36 cytokines were strongly expressed under these conditions and were readily purified as soluble proteins from the supernatant fraction of sonicated bacterial cell lysates. Proteins were washed and eluted from nickel‐NTA affinity matrix and yields were estimated at ~10 μg·mL^−1^ of bacterial culture.

### Processing of DEVD‐modified IL‐36 by purified caspase‐3

**Figure 3:**
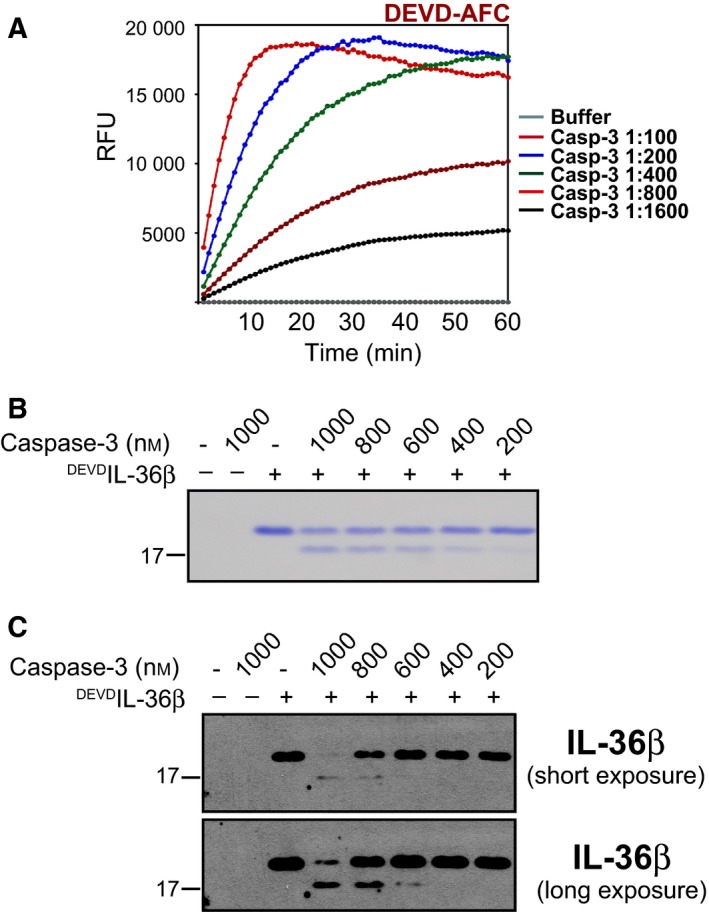
Caspase‐3 cleaves DEVD‐modified IL‐36. (A) Hydrolysis of the synthetic substrate Ac‐DEVD‐AMC (50 μm) by recombinant caspase‐3 was monitored over the indicated time‐course. (B) Recombinant full‐length ^DEVD^IL‐36β (100 ng·mL^−1^) was incubated at 37 °C for 2 h, either alone or in the presence of indicated concentrations of recombinant caspase‐3. Proteolysis was analysed by SDS/PAGE gel electrophoresis. (C) Full‐length ^DEVD^IL‐36β (100 ng·mL^−1^) was incubated at 37 °C for 2 h, either alone or in the presence of indicated concentrations of recombinant caspase‐3, followed by analysis by immunoblot.

To facilitate processing of DEVD‐modified IL‐36 cytokines, we expressed and purified recombinant caspase‐3 as previously reported 25. Purified caspase‐3 was highly active as demonstrated by its ability to cleave the synthetic caspase substrate DEVD‐AFC (Fig. 3A). We next incubated DEVD‐modified IL‐36α, β and γ with recombinant caspase‐3 over a range of concentrations from 1 μm to 200 nm and, as shown in Fig. 3B,C, this resulted in cleavage of the DEVD‐modified form of IL‐36β (^DEVD^IL‐36β) to a slightly smaller product consistent with removal of the N‐terminal residues as desired. Similar caspase‐3 cleavage reactions were also conducted with the DEVD‐modified forms of IL‐36α and IL‐36γ, with identical results (data not shown).

### DEVD‐modified IL‐36 cytokines are biologically active

**Figure 4:**
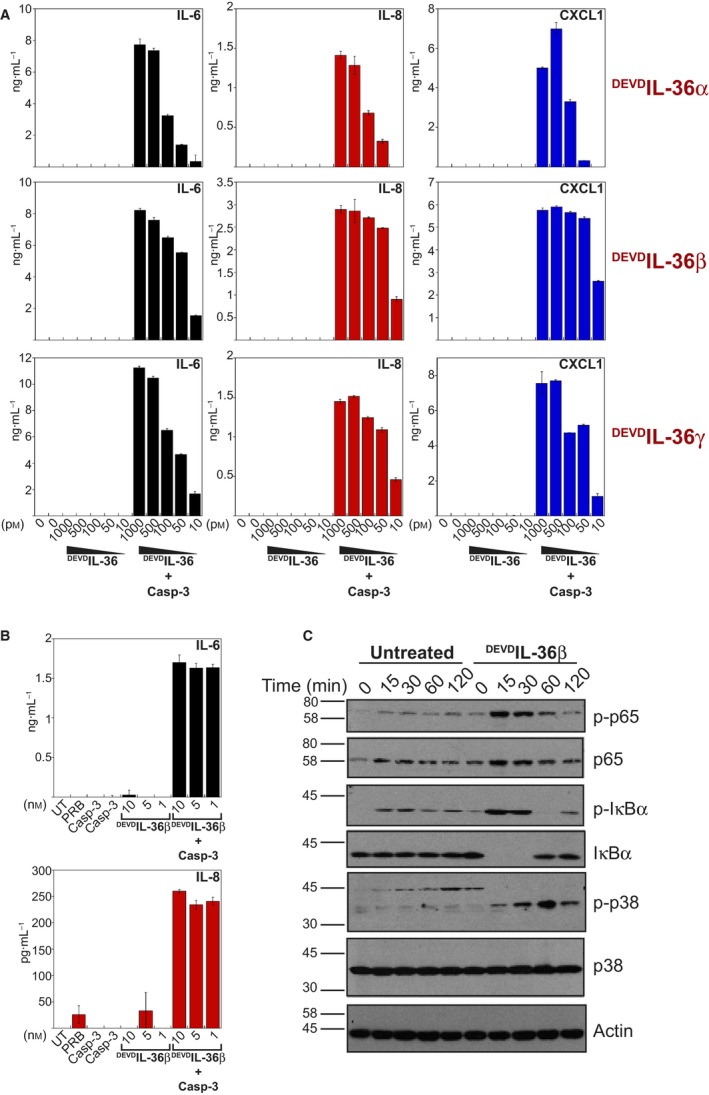
Truncated IL‐36 proteins exhibit biological activity. (A) ^DEVD^IL‐36α, ^DEVD^IL‐36β and ^DEVD^IL36γ (250 nm) were incubated either alone or in the presence of caspase‐3 for 2 h at 37 °C. HeLa cells stably overexpressing IL36R (5 × 10^4^ cells per well) were stimulated with a titration of ^DEVD^IL‐36, as indicated. After 18 h, cytokine concentrations in the culture supernatants were determined by ELISA. (B) ^DEVD^IL‐36β (5 mm) was incubated either alone or in the presence of caspase‐3 for 2 h at 37 °C. Protease reaction buffer (PRB) or caspase‐3 alone were used as negative controls. HeLa cells stably overexpressing IL36R (5 × 10^4^ cells per well) were stimulated with a titration of ^DEVD^IL‐36, as indicated. After 18 h, cytokine concentrations in the culture supernatants were determined by ELISA. (C) HeLa^IL36R‐SEAP^ cells (1 × 10^6^ cells per 6 cm plate) were stimulated, or not, with 5 nm active ^DEVD^IL‐36β. At indicated timepoints, cell lysates were made in 200 μL SDS loading buffer. Whole‐cell lysates were analysed by immunoblot with the indicated antibodies. Error bars represent the mean ± SEM of duplicate determinations from a representative experiment.

To ask whether caspase‐3‐cleaved DEVD‐modified IL‐36 cytokines were biologically active, we titrated full‐length versus caspase‐3‐cleaved forms of ^DEVD^IL‐36 α, β and γ onto HeLa cells that were stably transfected with the human IL‐36 receptor. As shown in Fig. 4A, whereas all three full‐length ^DEVD^IL‐36 cytokines were completely inactive in their unprocessed forms, processing of ^DEVD^IL‐36 cytokines with caspase‐3 resulted in highly robust biological activity as demonstrated by the production of very high levels of IL‐6, IL‐8 and CXCL1 by HeLa^IL‐36R^ cells. Importantly, caspase‐3 alone had no effect on cytokine production by HeLa cells (Fig. 4B), excluding the unlikely possibility that addition of the latter protease to the IL‐36 preparations could be the source of increased cytokine activity. We also examined some of the intracellular signal transduction events seen in response to active IL‐36. As shown in Fig. 4C, treatment of HeLa^IL‐36R^ cells with biologically active IL‐36 resulted in the rapid phosphorylation and proteolytic processing of the NFκB inhibitor IκB, in tandem with phosphorylation of the p65 subunit of NFκB, as well as phosphorylation of p38MAPK. Collectively, the above data clearly demonstrate that removal of the N‐terminal region of IL‐36 cytokines through insertion of a tetrapeptide caspase cleavage motif permitted the simultaneous removal of the poly‐histidine purification tag as well as the N‐terminal region responsible for autoinhibition of IL‐36 family cytokines, leading to the generation of highly active IL‐36.

### Generation of a reporter cell line for the assessment of IL‐36 activity

**Figure 5:**
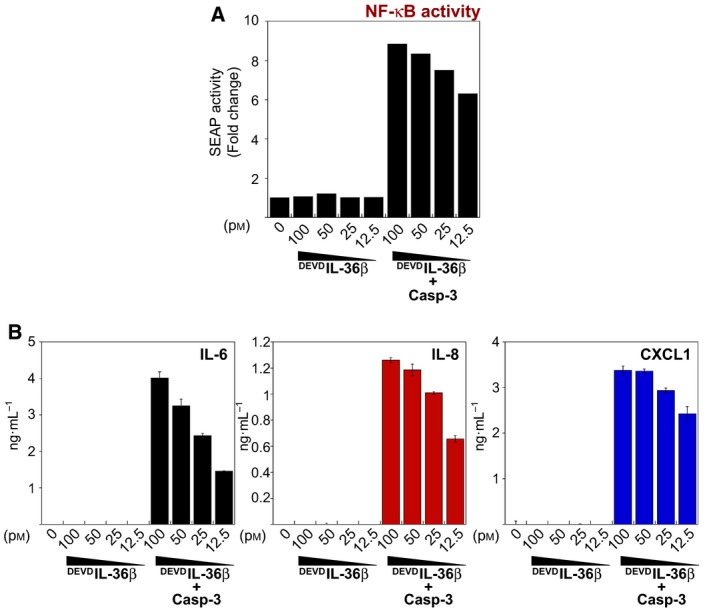
Truncated IL‐36 proteins activate NFkB. (A) HeLa^IL36R^ cells stably expressing the NF‐κB SEAP reporter plasmid were stimulated with a titration of ^DEVD^IL‐36β, as indicated. After 18 h, cell culture supernatant was incubated with Quantiblue reagent to detect secreted alkaline phosphatase in the medium. Absorbance was read at 620 nm after 4 h incubation with Quantiblue reagent. (B) Cytokine concentrations in the culture supernatants from (A) were determined by ELISA. Error bars represent the mean ± SEM of duplicate determinations from a representative experiment.

Because IL‐36 promotes NFκB activation (Fig. 4C), we also generated a convenient reporter cell line, based upon the NFκB‐dependent expression of secreted alkaline phosphatase (SEAP), to assess IL‐36 activity. As shown in Fig. 5A, HeLa^IL‐36R^ cells stably transfected with the NFκB‐SEAP reporter exhibited robust SEAP activity upon treatment with caspase‐3‐cleaved ^DEVD^IL‐36. As expected, HeLa^IL‐36R‐SEAP^ cells also secreted multiple cytokines in response to IL‐36 treatment (Fig. 5B). This reporter line will be useful for future studies aimed at identifying upstream components of IL‐36 signal transduction events and also for screening of inhibitors of IL‐36 signalling.

### Transformed and primary keratinocytes are responsive to IL‐36

**Figure 6:**
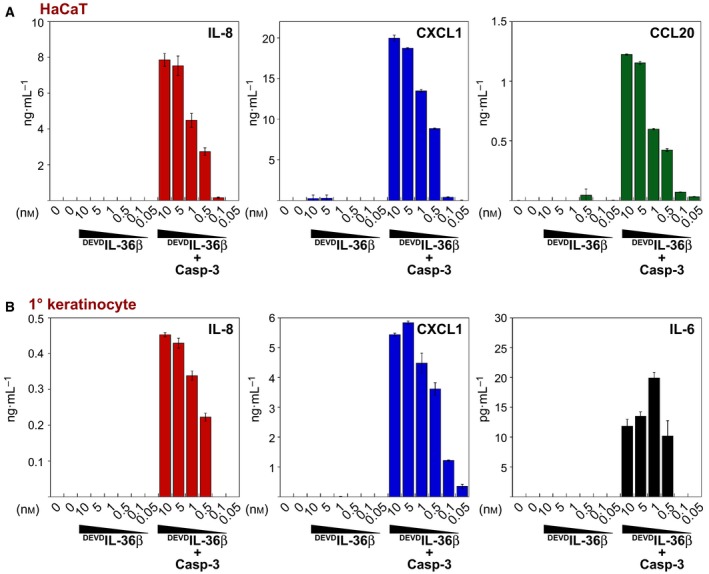
Truncated IL‐36 proteins exhibit biological activity on primary and transformed keratinocytes. (A) ^DEVD^IL‐36β (250 nm) was incubated with caspase‐3 (400 nm) for 2 h at 37 °C. HaCaT cells (5 × 10^4^ cells per well) were stimulated with ^DEVD^IL‐36β, as indicated. After 18 h, cytokine concentrations in the culture supernatants were determined by ELISA. (B) Primary human keratinocytes (4 × 10^4^ cells per well) were stimulated with ^DEVD^IL‐36β, as indicated. After 18 h, cytokine concentrations in the supernatants were determined by ELISA. Error bars represent the mean ± SEM of duplicate determinations from a representative experiment.

Because the preceding tests of the biological activity of IL‐36 cytokines were conducted on cells overexpressing the IL‐36 receptor, we also asked whether cells that naturally express the IL‐36R were IL‐36 responsive. As noted in the Introduction, IL‐36 is strongly linked with psoriasis which is thought to result from excessive inflammation in the epidermal layers of skin, driven in part through the release of inflammatory mediators from keratinocytes. Thus, we asked whether transformed HaCaT keratinocytes, as well as primary human keratinocytes were responsive to caspase‐3‐cleaved DEVD‐modified IL‐36 cytokines. As shown in Fig. 6, both cell types were highly responsive to caspase‐3‐cleaved, but not full‐length, ^DEVD^IL‐36, once again confirming that the former is highly active and can be readily produced in large amounts for the exploration of IL‐36 function in cellular and molecular studies. Furthermore, these data also show that insertion of a simple tetrapeptide caspase cleavage motif is a useful strategy for the facile removal of purification tags or protein domains that interfere with protein solubility or activity.

### Generation of polyclonal antibodies against individual IL‐36 proteins

**Figure 7:**
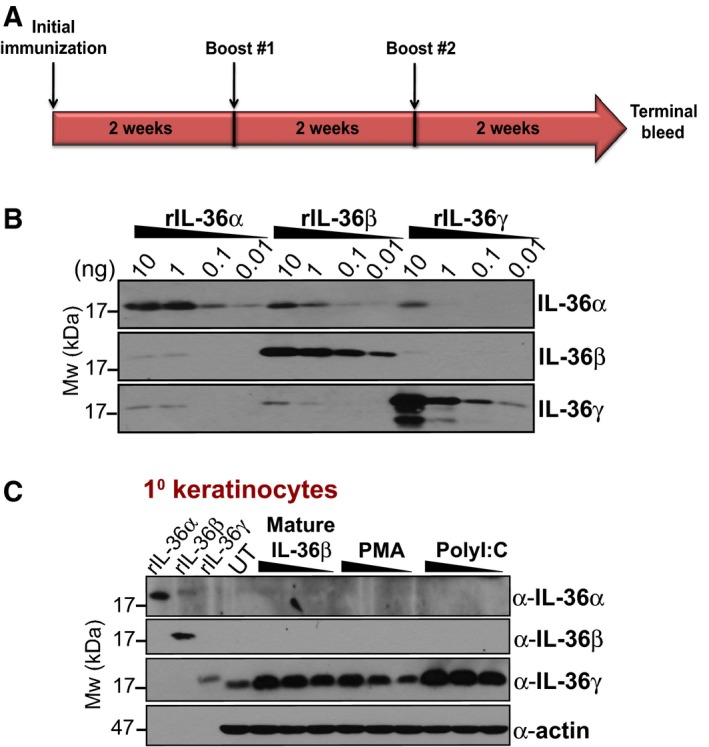
Generation of polyclonal antibodies against human IL‐36 proteins. (A) Schematic of the immunization protocol for generation of IL‐36 polyclonal antibodies. (B) Western blot analysis of the specificity of IL‐36 polyclonal antibodies tested against indicated amounts of recombinant IL‐36 proteins. (C) Primary keratinocytes were treated with a titration of mature IL‐36β (10, 5, 2.5 nm), PMA (40, 20, 10 nm) and Poly(I:C) (100, 50, 25 μg·mL^−1^). Lysates were prepared and analysed by immunoblot using IL‐36 polyclonal antibodies described in (B). Recombinant IL‐36 (500 pg) serves as a positive control for each immunoblot.

Finally, we also used recombinant IL‐36 proteins to immunize rabbits with individual IL‐36 proteins (i.e. α, β or γ) to generate polyclonal antibodies capable of recognizing the latter cytokines (Fig. 7A). As Fig. 7B illustrates, this approach yielded antibodies that were highly specific for IL‐36β and IL‐36γ, with little cross‐reactivity against each other, as well as antibodies that were relatively specific for IL‐36α. Moreover, the IL‐36γ polyclonal antibody was capable of detecting endogenous levels of IL‐36γ from primary human keratinocytes (Fig. 7C). These antibodies will be useful for investigations exploring the factors that influence IL‐36 expression in healthy versus inflamed tissues.

## Discussion

Here, we have shown that the insertion of a caspase‐3 cleavage motif (DEVD) into IL‐36 cytokines enabled us to produce highly active and soluble forms of these cytokines upon processing with caspase‐3. These data also confirmed that proteolytic processing of IL‐36α, β and γ dramatically increase the biological activity of these cytokines. We also established a HeLa^IL‐36R^ bioassay to screen for proteases that naturally activate IL‐36 cytokines.

IL‐36 cytokines require N‐terminal processing for activation, however the proteases responsible for this activation are unknown. Thus, the insertion of DEVD motifs allowed us to potently activate IL‐36 cytokines using caspase‐3 to generate truncations at sites previously identified to liberate biological activity within these cytokines 22. Caspases have exquisite specificity for cleavage after aspartic acid residues and so can selectively and efficiently process the DEVD tetrapeptide which is not commonly found in many proteins, especially within surface exposed loop regions prone to proteolysis. The DEVD peptide was originally identified from the caspase cleavage site within PARP, a DNA repair enzyme, targeted by caspase‐3 during apoptosis 27. Derivatives of DEVD are frequently used to assess caspase‐3 enzymatic activity by fluorimetric methods and also as inhibitors of caspase‐3 activity. Here, we have demonstrated a novel use of the DEVD motif as a fusion tag to generate proteins that are susceptible to proteolysis by caspase‐3. Similar strategies have been previously used by other groups to purify biologically active forms of IL‐36RA and IL‐38 22, 28. IL‐36RA requires removal of the N‐terminal Met1 residue to act as an antagonist of IL‐36 signalling but exhibits little activity when this residue is present 22. Towne *et al*. used a factor Xa protease removable N‐terminal GST tag to generate truncated IL‐36RA. However, one potential drawback of the latter approach is that GST, being a relatively large fusion partner (26 kDa), may introduce solubility and folding problems in proteins tagged using this strategy. Furthermore, removal of the GST tag by factor Xa has been reported to be relatively ineffective and nonspecific, reducing yields of the desired cleavage product 29, 30.

Small ubiquitin‐related modifier (SUMO) fusion tags have also been used to purify N‐terminally truncated IL‐36 ligands 22. SUMO is a relatively small protein tag (~11 kDa) often used as a solubility enhancer for protein purification by promoting proper folding and stability. After purification, the SUMO moiety can be removed by the highly specific SUMO protease UlpI. However, unlike the GST tag, SUMO requires an additional affinity tag for purification of the fusion protein, usually a polyhistidine tag 30. Hu *et al*. recently described the purification of IL‐38 as a TEV fusion protein. TEV protease, from the tobacco etch virus, is more stringent than factor Xa and other commonly used fusion tag proteases like thrombin and enterokinase. Wild‐type TEV protease can be deactivated by autolysis and expression of the protease is hindered by solubility issues, however, mutated forms of the protease have been developed for increased stabilization and expression yields 31. Inclusion of the small caspase‐3‐specific peptide tag DEVD acts as a simple and reliable alternative for accurate removal of purification tags and inhibitory domains within proteins with minimal disruption to the fusion partner.

We also established a convenient reporter cell line HeLa^IL‐36R^, which overexpresses the NFκB‐responsive SEAP reporter plasmid. This bioassay permits the rapid screening of compounds that naturally antagonize IL‐36 cytokine activity. Activation of NFκB signalling by IL‐36 induces the release of secreted alkaline phosphatase into culture medium that is highly stable and can be readily detected by colorimetric assay. This allows the rapid and continuous monitoring of NFκB activation without the need to disrupt or lyse cell populations.

It is interesting that removal of very small numbers of residues from the N‐termini of IL‐36 family cytokines have such dramatic effects on the biological activities of these cytokines. For example, the IL‐36 N‐termini may partly occlude the receptor binding domain through steric clashes, removal of which permits a more stable interaction with the IL‐36R complex. Alternatively, proteolysis of IL‐36 cytokines may induce a conformational change in these proteins that increases their affinity for the IL‐36 receptor, thereby increasing biological potency. Interestingly, Hazuda and colleagues have reported that IL‐1α and IL‐1β cytokines undergo profound conformational changes upon removal of their N‐termini and, as a consequence, the mature regions of these molecules switch from a proteinase K‐sensitive to a proteinase K‐insensitive state 32. This change is most likely reflected in an altered conformation that increases affinity for the IL‐1 receptor 32. The further resolution of IL‐36 ligand structures, both in their full‐length, as well as their processed forms by naturally occurring proteases, will help to elucidate how exactly these molecules interact with the IL‐36R/IL‐1AcP receptor complex.

In conclusion, here we have described a novel method to produce highly soluble and active recombinant IL‐36 cytokines through insertion of a small caspase cleavage motif within the N‐terminal region of these cytokines, followed by processing by caspase‐3. IL‐36 cytokines have been reported to have an important role in the pathogenesis of psoriasis, a common skin inflammatory condition. We have shown both transformed and primary keratinocytes, which naturally express IL‐36R, were highly responsive to active caspase‐3‐cleaved DEVD‐modified IL‐36. This method facilitates production of active IL‐36α, IL‐36β and IL‐36γ that can be used to further investigate the role of the IL‐36 subfamily in inflammation.

## Experimental procedures

### Materials

Anti‐IL‐36β antibody was purchased from R&D (Minneapolis, MN, USA) systems. Antibodies specific to p‐p65, p‐p38 and p38 were obtained from Cell Signalling Technology (Danvers, MA, USA). Anti‐p65 and anti‐IκBα were from Santa Cruz Biotechnology (Dallas, TX, USA). Anti‐p‐IκBα was from BD Pharmingen (Oxford, UK) and anti‐actin was from MP Biomedicals (Santa Ana, CA, USA). Polyclonal antibodies were generated against IL‐36α, β and γ proteins by repeated immunization of rabbits with the full‐length recombinant IL‐36 proteins (Biogenes, Berlin, Germany). Synthetic peptide Ac‐DEVD‐AFC was purchased from Bachem (Weil am Rhein, Germany). QuantiBlue reagent for detection and quantification of secreted alkaline phosphatase was from InvivoGen (San Diego, CA, USA). Unless otherwise indicated, all other reagents were purchased from Sigma (Arklow, Ireland).

### Cell culture

The HeLa^IL36R^ cell line was generated by transfection with pCXN2.IL‐1Rrp2 (IL‐36R) plasmid followed by selection using G418 antibiotic (Sigma). IL‐36R overexpressing clones were expanded from single cells using limiting dilution cloning, followed by expansion of individual clones. The HeLa^IL36R‐SEAP^ cell line was generated by transfection of stable HeLa^IL36R^ overexpressing cells with pNifty2‐SEAP plasmid (InvivoGen) followed by selection using Zeocin antibiotic. Clones were expanded from single cells using limiting dilution cloning. Clones were selected by demonstration of acquired optimal responsiveness to active forms of IL‐36 via SEAP production and ELISA. HaCaT cells were cultured in Dulbecco's modified eagle's medium (DMEM) (Gibco, Waltham, MA, USA) supplemented with FCS (10%). Primary neonatal foreskin‐derived keratinocytes were purchased from Cell Systems (Troisdorf, Germany) and cultured in serum‐free Dermalife K media (Cell Systems). All cells were cultured at 37 °C in a humidified atmosphere with 5% CO_2_.

### Expression and purification of recombinant ^DEVD^IL‐36 and caspase‐3

Modified forms of IL‐36 containing a caspase‐3‐processing motif (DEVD) N‐terminal to known processing sites 22 were generated by cloning the DEVD‐modified cytokine coding sequence in frame with the poly‐histidine tag sequence in the bacterial expression vector pET45b. Proteins were expressed by addition of 600 μm IPTG to exponentially growing cultures of BL21‐CodonPlus(DE3)‐RIL bacteria followed by incubation for 3 h at 37 °C. Bacteria were lysed by sonication and poly‐histidine tagged proteins were captured using nickel‐NTA agarose (Amintra, Expedeon Ltd, Cambridgeshire, UK), followed by elution into PBS, pH 7.2, in the presence of 100 mm imidazole. Recombinant poly‐histidine‐tagged caspase‐3 was expressed and purified as described previously 25. Bacterial cell lysates and purified proteins were visualized on 12% SDS/PAGE elecrophoresis gel stained with Coomassie Brilliant Blue.

### Caspase‐3 activity assay

Protease cleavage reactions (40 μL final volume) were carried out in protease reaction buffer (50 mm HEPES, pH 7.4, 75 mm NaCl, 0.1% CHAPS, 2 mm DTT) containing 50 μm Ac‐DEVD‐AFC. Samples were measured by using an automated fluorimeter (Spectrafluor Plus; TECAN, Reading, UK) at wavelengths of 430 nm (excitation) and 535 nm (emission) for 60 min.

### Caspase‐3 cleavage assays

Reactions were carried out in protease reaction buffer (50 mm HEPES, pH 7.4, 75 mm NaCl, 0.1% CHAPS, 2 mm DTT) for 2 h at 37 °C. Full‐length or processed ^DEVD^IL‐36 cytokines were then used to treat cells (5 × 10^4^ cells per well) for 18–24 h, followed by assessment of cytokine production by ELISA.

### Measurement of cytokines and chemokines by ELISA

Cells were plated at 5 × 10^4^ cells per well and treated with various forms of IL‐36 as indicated in the appropriate figure legends. Cytokines and chemokines were measured from cell culture supernatants using specific ELISA kits obtained from R&D Systems (human IL‐6, IL‐8, CXCL1, CCL20). All cytokine assays were carried out using duplicate samples from each culture.

### Measurement of NFκB activation by SEAP assay

Hela^IL36R‐SEAP^ cells were plated at 5 × 10^4^ cells/well in a tissue culture dish in RPMI supplemented with 5% heat‐inactivated FCS. Cells were treated with a titration of full‐length or caspase‐3‐activated ^DEVD^IL‐36β. Cell culture supernatants were harvested and secreted alkaline phosphatase in the medium was detected using Quantiblue reagent (InvivoGen). Ten microlitres of cell culture supernatant was incubated with 190 μL prewarmed QuantiBlue reagent and incubated for 15 min at 37 °C. To assess SEAP activity, absorbance readings were taken at 620 nm with a microplate reader (Infinite F50; TECAN) after 15 min to 6 h incubation periods.

### Western immunoblotting

Protein samples were prepared using SDS/PAGE loading buffer (2% SDS, 50 mm Tris–HCl, pH 6.8, 10% glycerol, 2.5% β‐mercaptoethanol), boiled for 7 min and electrophoresed on 12% SDS/PAGE gels. Proteins were then transferred onto nitrocellulose membrane at 40 mA overnight. Membranes were blocked for 1 h (5% NFDM, 0.05% sodium azide in Tris‐buffered saline, Tween‐20, TBST). The indicated proteins were probed using specific antibodies, typically diluted 1 : 1000. Membranes were washed 3 times in TBST and then incubated with the relevant HRP‐conjugated secondary antibody diluted 1 : 2000. Membranes were again washed and proteins were visualized with SuperSignal West Pico (Thermo Scientific, Waltham, MA, USA) and exposure to autoradiography films.

## Author contributions

D.M.C. and C.M.H. performed experiments, analysed data, generated the figure panels and wrote the figure legends. G.P.S., P.B.D. and E.B. purified recombinant proteins and validated some of the cell line data. S.J.M conceived the study, designed and analysed experiments, supervised the study and wrote the manuscript with contributions from D.M.C. and C.M.H.
